# Practical recommendations for strengthening national and regional laboratory networks in Africa in the Global Health Security era

**DOI:** 10.4102/ajlm.v5i3.471

**Published:** 2016-10-31

**Authors:** Michele Best, Jean Sakande

**Affiliations:** 1 Corporate Director of Laboratories, Dimensions Healthcare System, Cheverly, Maryland, United States; 2 Laboratory of Biochemistry, University of Ouagadougou, Ouagadougou, Burkina Faso

## Abstract

The role of national health laboratories in support of public health response has expanded beyond laboratory testing to include a number of other core functions such as emergency response, training and outreach, communications, laboratory-based surveillance and data management. These functions can only be accomplished by an efficient and resilient national laboratory network that includes public health, reference, clinical and other laboratories. It is a primary responsibility of the national health laboratory in the Ministry of Health to develop and maintain the national laboratory network in the country. In this article, we present practical recommendations based on 17 years of network development experience for the development of effective national laboratory networks. These recommendations and examples of current laboratory networks, are provided to facilitate laboratory network development in other states. The development of resilient, integrated laboratory networks will enhance each state’s public health system and is critical to the development of a robust national laboratory response network to meet global health security threats.

## History of laboratory networks in Africa

Efforts to combat HIV, tuberculosis and malaria in sub-Saharan Africa in the past decade have underscored the inadequacy of laboratory systems in most African countries, as they were not equipped to perform vital testing for HIV care and prevention programmes. Laboratory testing is required for the diagnosis and management of these diseases and many other communicable and non-communicable diseases endemic to the African region. For many years, there had been steady neglect of national health laboratories in Africa, leading to a lack of trust in laboratory results and empiric treatment of patients. Early improvement efforts employed a vertical approach, creating national HIV- and AIDS-specific laboratories. This approach has proved inadequate, as it created competition for resources and did not improve the entire laboratory system in the countries.^1^ It became clear that all laboratories in a country should be part of a fully-integrated laboratory network that can address both individual patient care needs and public health needs from the peripheral levels to the national level.

In the past decade, there has been a focus on the development of a national laboratory network that is sustainable and will be more efficient in the use of limited resources. In 2008, a number of international meetings helped to focus efforts on an integrated network approach to laboratory system strengthening efforts. These included the Maputo Declaration in Mozambique^2^ and the World Health Organization’s Regional Office for Africa (WHO AFRO) Resolution AFR/RC/58/R2, which was issued during the 58th session of the Regional Committee held in Yaoundé, Cameroon, in September 2008.^3^ The creation of the Stepwise Laboratory (Quality) Improvement Process Towards Accreditation System by WHO AFRO^4^ and the Strengthening Laboratory Management Toward Accreditation programme in 2009 were major steps toward quality improvement of laboratories in the region.^5^ These actions led to the development of National Laboratory Strategic Plans and a National Laboratory Policy in numerous countries, including Botswana, Burkina Faso, Eritrea, Ethiopia, Cameroon, Kenya, Liberia, Mali, Mauritius, Senegal and Tanzania. One focus of these countries has been the development of an integrated, tiered national public health laboratory network that can provide high-quality, accessible and efficient laboratory testing services for the entire population.

There are many examples of functional laboratory networks in the African region. The Polio Laboratory Network in African region has played a critical role in diagnosing poliovirus disease (poliomyelitis) and the detection of poliovirus transmission, resulting in many countries in Africa having been declared free of polio.^6^ WHO AFRO supports a public health laboratory network, including national reference centres, sub-regional and regional reference laboratories for specific diseases and WHO collaborating centres for plague. This laboratory network-based surveillance of meningitis epidemics has played a significant role in the timely outbreak response in meningitis-belt countries.^7^ In response to the Maputo Declaration, a number of sub-regional networks dedicated to disease surveillance, prevention and response have been launched in Africa with the support of several international development partners. One partner, the West African Laboratory Network, funded by the French Development Agency and Foundation Mérieux in 2009, focuses on three main areas of activity: training laboratory personnel, setting quality assurance, and strengthening epidemiological surveillance in seven West African francophone countries, including Guinea, since 2013. The West African Laboratory Network allowed for the first diagnosis of Ebola virus disease by the Jean Merieux P4 Laboratory in Lyon.^8^ Another partner, the East Africa Public Health Laboratory Network Project, includes three mutually-reinforcing components which are supporting the five East Africa Community member states to diagnose communicable diseases of public health importance and to share information about those diseases to mount an effective regional response.^9^ In addition, effective national laboratory networks have been developed in a number of countries, including Ethiopia and Tanzania, as a result of the Maputo Declaration^2^ and Resolution AFR/RC58/R2.^3^

Despite the above exemplary achievements in developing laboratory networks, the 2014–2015 Ebola virus disease outbreak in West Africa highlighted gaps and unmet needs in these countries and underscored the importance of redoubling disease prevention and control efforts in Africa. While many countries have made progress in strengthening diagnostic and surveillance capacity, much more needs to be done, particularly to comply with the 2005 WHO International Health Regulations.^10^ Consequently, there is a great need for a gap analysis assessment of current laboratory systems in Africa to promote innovative approaches to achieving sustainable national laboratory networks throughout Africa. To this end, a laboratory network assessment tool, the LABNET scorecard, was developed by the African Society for Laboratory Medicine to assist countries in assessing the development of their networks.^11^

## The Global Health Security Agenda and National Laboratory Networks

The Global Health Security Agenda was launched in February 2014 by a group of nations working collaboratively to promote global health security as an international priority. It is led by a Global Health Security steering group of 10 member nations. The goal of the agenda is to accelerate progress toward a world safe and secure from global health threats posed by infectious diseases. Diseases of recent global threat concern include Ebola virus disease, Middle East respiratory syndrome, avian influenza, yellow fever, and multi-drug resistant tuberculosis. In May 2014, a Global Health Security Commitment Development meeting was held in Helsinki and identified 11 Global Health Security Action Packages, five of which have a laboratory component,^12^ including Antimicrobial Resistance, Zoonotic Disease, Biosafety and Biosecurity, Immunization, and National Laboratory System. The goal of the National Laboratory System package is the development of safe and secure laboratory capacity in each country. It contains an action item to evaluate and improve capacity at national reference, provincial and district laboratories for 10 core laboratory tests. The core tests include bacterial culture for *Salmonella enteritidis* serotype typhi, viral culture for poliovirus, PCR for influenza virus, serology for HIV, microscopy for *Mycobacterium tuberculosis*, and rapid diagnostic testing for *Plasmodium* spp., as well as at least four tests selected by the country on the basis of its major public health concerns. The five-year target is real-time biosurveillance with a national laboratory system and effective modern point-of-care and laboratory-based diagnostics. It is expected that the laboratory system will be able to reliably conduct testing on at least five of the 10 core tests on specimens transported to accredited laboratories from at least 80% of the districts in the country.^12^ In addition, laboratories must develop plans for detection and surveillance of antimicrobial resistance and surveillance of zoonotic diseases.

In summary, there is a need to establish functional national laboratory networks for various reasons, including to provide laboratory confirmation for priority diseases, support national integrated disease surveillance, provide individual patient care, and implement various aspects of the Global Health Security Agenda. Accurate laboratory data are essential for clinicians to accurately assess the status of patients’ health, make accurate diagnoses, formulate treatment plans, and subsequently monitor the effects of treatment. This applies to both communicable and non-communicable diseases. A tiered, integrated laboratory network with strong supporting core capabilities will provide the best model for efficient service delivery across various levels of the public health system.^13^

Traditionally, the public health laboratory has been the sole response laboratory for public health emergencies; however, a new model has emerged,^14^ exemplified by the US Centers for Disease Control and Prevention’s Laboratory Response Network, a national network of laboratories charged with identifying and characterising agents of bioterrorism and other threats to public health. In this new model, the national public health laboratory leads a network of clinical and other agency laboratories, such as food testing, veterinary, and local public health laboratories, to support public health response.

## Major requirements for functional national laboratory networks

### Effective implementation of National Strategic Plans

The development of an integrated, functional, high-quality public health laboratory network requires the development of a National Laboratory Strategic Plan to provide the vision and roadmap for its implementation. A guidance document to assist countries in development of National Laboratory Strategic Plans was published by the WHO and US Centers for Disease Control and Prevention in 2008.^15^ This document, and samples of Strategic Plans from other countries, may be helpful in the development of a tiered laboratory network. Each country must develop its own plan as it will be unique to its existing medical, laboratory and regulatory structure.

The initial step in the development of the Strategic Plan is the appointment of a leadership team or working group by the country’s Ministry of Health that possesses deep knowledge of the current national laboratory situation.^16^ As part of strategic planning, the group will assess the current state of laboratories in the country with an assessment of strengths and weaknesses using the African Society for Laboratory Medicine’s LABNET scorecard for assessing the national laboratory network functionality.^11^ As part of the strategic planning process, leadership will determine priorities for strengthening the national laboratory network system and develop a vision, goals, objectives, expected results and strategies for the next three to five years to achieve the desired results. Using the available resources and partners’ support, the working group will finalise the strategic/implementation plan with an assigned budget and time-frames. The working group should present the finalised Strategic Plan to Ministry of Health policy makers and planning authorities for approval. If approved, this plan should be incorporated into the health sector plan. Following the approval of the Strategic Plan, obtaining essential resources, and preparation of the operational guide or a well-formulated constitution, a formational meeting should be organised. The next step is to launch the national laboratory network and begin to implement the plan.

It is important to note that a Strategic Plan is not an implementation plan – it merely provides the strategic initiatives on which the implementation plan for the development of the laboratory network is based. Based on the Strategic Plan initiatives, a variety of planned activities will be needed to address gaps in current systems. Activities must be prioritised as certain activities are required in order for others to take place. For example, a commodities list cannot be developed until the equipment plan for the network is finalised. An implementation plan must be developed with action plans for activities that are required to accomplish key strategies. Estimated costing information is required for all activities in the implementation plan for the first three years, at a minimum. Adequate funding of the plan is essential for its success. Ideally, an implementation team would be led by assigned laboratory leadership and a technical working group in consultation with implementation consultants. Implementation of the plan is the most critical and challenging aspect of the strategic planning process. Top laboratory leadership will need to own the implementation plan and monitor progress regularly. Plan implementation requires a separate monitoring and evaluation plan to determine whether planned targets are being met; Strategic Plans must be reviewed at least annually for progress made toward objectives. The plan may need to be updated as circumstances change or at regular intervals.

### Adequate financial support

As part of the strategic and implementation plan, cost estimates must be developed for all activities in the plan. This will provide Ministry of Health officials with anticipated costs of the laboratory network development for the next three to five years. Costs should include laboratory renovation, equipment, human resources, reagents and supplies, quality assurance, external quality assessment and accreditation, specimen referral, training, and other costs. An annual budget for the national laboratory network should be developed by the Director of the Division of Laboratories and approved by the Ministry of Health as part of the health sector budget process. The budget should be adequate to cover the annual capital and operating expenses of the laboratory network.

### A national laboratory policy and regulatory framework

As part of the health sector policy for the country, a national laboratory policy provides the regulatory framework for the laboratory network within the country. National laboratory standards are developed that apply to all laboratories operating in the country, including public, private, and mission-based laboratories. These standards cover a variety of areas, including human resources, infrastructure, equipment, in vitro diagnostic device (IVD) regulation, and analysis/activities by health system level. It should be aligned with the International Health Regulation and Global Health Security Agenda requirements.

### Integrated, tiered national laboratory network development

**FIGURE 1:**
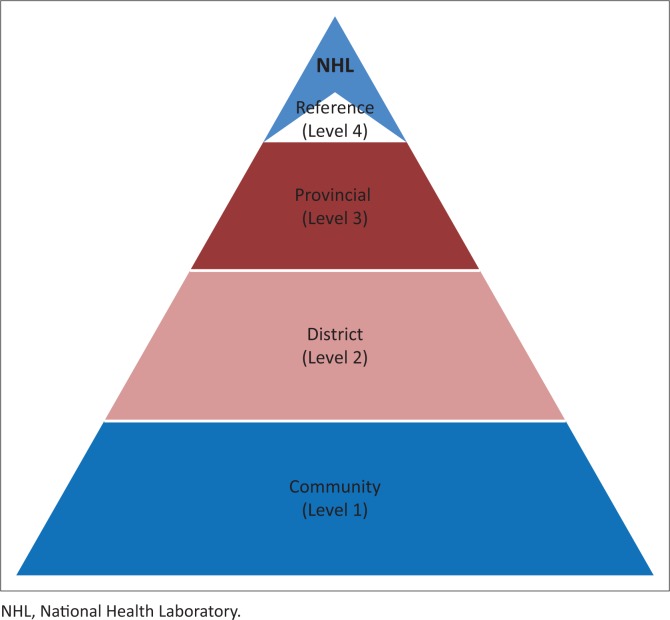
The integrated Tiered Laboratory Network (Level 4 in some countries includes the reference laboratories and the National Public Health Laboratory is on top of the system).

An integrated, tiered national laboratory network is an integrated system of laboratories organised in three or four tiers and aligned with the public health delivery system of the country (Figure 1).^17^ Levels of laboratories are determined by their test menus and functions, and a referral network is established in order to perform tests at the most appropriate level of the tiered system. The test menu available at each tier or level of the laboratory network would depend on infrastructure available and testing needs for patients. An example of test menus for a tiered network is shown in Table 1, and an example of a grid mapping different details of key requirements by each tier of the network is shown in Table 2. Point-of-care and rapid test kits should be chosen carefully and used wherever possible to allow for ease of use and reliability. Testing would be referred up the pyramid of laboratories with communications going both up and down. The number of tiers in the laboratory system and the test menu performed at each level may vary depending on service level needs, priority diseases, geographic coverage, referral capabilities, and resources available. It may be desirable to first prioritise the full development of the national public health laboratory (Tier 4) and a good referral system, as the national reference laboratory could initially serve the testing needs for the country while the rest of the network is being developed. A plan for the geographic location and development of all laboratories in the network would be the next priority in the plan.

**TABLE 1:** Example of laboratory tests and services needed at each level of a tiered national laboratory network.

Laboratory Tier	Proposed Test Menu/Functions
Community Health Services (Tier 1)	Haemoglobin HIV Rapid Test (HIV1/2) Malaria/Blood Parasite Smear Rapid Malaria Test Rapid Syphilis Test Urine Dipstick for sugar and protein Specimen collection for referral to higher tier laboratories Preparation of DBS for DNA PCR
District Laboratory (Tier 2)	All tests listed for Level 1 In addition: AFB/TB Smear CBC with automated differential CD 4 (Point of Care Test) Basic Chemistry Panel with ALT and Creatinine CSF/Body Fluid and urine microscopy Cryptococcus Antigen and/or India Ink GeneXpert Rapid TB testing Gram Stain and Primary Culture Type and Cross-match Urine pregnancy test Wet mounts Viral Load (Point of care) Ebola RDT
Regional/Provincial Laboratory (Tier 3)	All tests listed in for Levels 1 and 2 In addition: Complete Chemistry Panel Microbiology Culture, full ID and AST Hepatitis B and C Antibody
National Health Laboratory (Tier 4)	All tests listed in for Levels 1, 2 and 3 In addition: HIV DNA PCR HIV ELISA AFB culture, ID, Sensitivity Ebola PCR PCR and Molecular Testing HIV Drug Resistance Testing Complex Confirmatory Testing Referral of testing to international reference laboratories

DBS, dried blood spot; AFB, acid-fast bacilli; TB, tuberculosis; CBC, complete blood count; CSF, cerebrospinal fluid; RDT, rapid diagnostic test; ID, identification.

**TABLE 2:** Example of mapping grid of laboratory requirements for different tiers.

Laboratory Requirement	Level 1	Level 2	Level 3	Level 4
Physical Structure	Basic laboratory with draw station or counter designed for lab testing	Laboratory design in one room with different benches with biosafety equipment	Larger laboratory design with separate lab testing areas and biosafety equipment	National Public Health Laboratory Structure
Biosafety Requirements	Basic blood and body fluid precautions (P1)	Biosafety Level 2 or 3 (P2 or P3), depending on menu	Biosafety Level 2 or 3 (P2 or P3), depending on menu	Biosafety Level 4 (P4)
Management/Staffing	Laboratory technicians or nurses trained in laboratory techniques	Laboratory technicians/ phlebotomist/ Laboratory Supervisor	Laboratory technicians/ Medical Technologists/ phlebotomist/Laboratory Manager	Medical technologists/ Laboratory Scientists/ Laboratory Director
Equipment/kits	Haemoglobin Meter RDTs (Malaria, HIV, Ebola, Syphilis…) Urine Dipsticks Sample collection kits/materials	Haematology analyser (low volume) Chemistry analyser (low volume) Microscope CD4 POCT GeneXpert Viral load POCT Ebola RDT RDTS and materials from Level 1	Higher volume Chemistry analyser (Higher volume) Hematology Analyser (higher volume) AST equipment (MIC) Microscope ELISA analyser CD4 analyser Ebola RDT GeneXpert Viral load POCT RDTs and materials from Level 1	Full Menu Chemistry Analyser Full Menu Haematology Analyser Full menu immunoassay analyser CD4 analyser PCR analyser Sequencer Microscan or Vitek (MIC)
EQA	HIV Malaria	Haematology Microbiology Chemistry CD4 Viral Load HIV Rapid Malaria smear Malaria RDT	Haematology Microbiology Chemistry CD4 Viral Load HIV Rapid Malaria Smear Malaria RDT	Microbiology PCR/Molecular Chemistry Haematology CD4 Viral Load
Accreditation	None	SLIPTA checklist	SLIPTA checklist	International accreditation
Transport Method	Trained and certified courier system	Trained and certified courier system	Trained and certified courier system	Certified courier, Fedex or air transport
Test/Specimen Referral Plan	Routine culture to Regional Lab; DBS for DNA PCR to National Public Health Lab	Full Microbiology ID referred to Regional lab; DNA PCR to national health lab.	Confirmation on unusual pathogens sent to National Health Lab; DNA PCR sent to national health lab	SARS or viral illness of international concern sent to global reference labs
Supply requirements	Standard commodities list for Level 1; reliable supply management and distribution system	Standard commodities list for Level 2; reliable supply management and distribution system	Standard commodities list for Level 3; reliable supply management and distribution system	Standard commodities list for Level 4; reliable supply management and distribution system
Communication /Laboratory Report	Fax and email, SMS	Fax and email, SMS	Hospital or laboratory information system	Laboratory Information system

RDT, rapid diagnostic test; POCT, point-of-care test; MIC, minimum inhibitory concentration; ELISA, enzyme-linked immunosorbent assay; SLIPTA, Stepwise Laboratory Quality Improvement Process Towards Accreditation; DBS, dried blood spot; ID, identification; SARS, severe acute respiratory syndrome.

Medical and public health laboratory services are a critical component of national health systems and are central to disease diagnosis, treatment, prevention, surveillance and outbreak investigations. When used optimally, laboratory testing generates knowledge that can facilitate patient safety, improve patient outcomes and lead to more cost-effective healthcare. In African countries, laboratory testing influences medical decision making in nearly half of the cases presenting to primary healthcare facilities. The vast majority of the population (except for the few who can afford private healthcare) depend on public health services for all their healthcare needs.

Laboratory services need to be fully integrated as a core component of health systems, yet very few countries in the African region have clearly defined the role of laboratory services at each level of the healthcare pyramid. Most countries are not aware of what laboratory services are being offered to the population in terms of types of tests and their quality. As a consequence, national planning for laboratory personnel and support services is almost non-existent.

In most countries, laboratory services are charged at subsidised prices and are a major income-generating unit in health facilities. However, much of the laboratory income is used to support other services, leaving the laboratory deficient in essential resources. There is an urgent need to re-examine the approach to formulating essential primary healthcare (district) laboratory packages to address the health problems in different local contexts, and to re-define issues of access, quality and how public laboratory services should be funded and sustained.

For a national laboratory network to be fully functional, it must be part of the country’s universal health coverage and embody three related objectives. The first objective is to ensure equity in access to health services; everyone who needs services should get them, not only those who can pay for them. The second objective is that the quality of health services should be good enough to improve the health of those receiving services. The third objective is that people should be protected against financial risk, ensuring that the cost of using services does not put people at risk of financial harm. For a national laboratory service to meet universal health coverage goals, countries must define how a primary healthcare laboratory package should be formulated to contribute to universal health coverage in African country settings, including formulating quality laboratory packages at all levels of the national health system, ensuring equity in access to the laboratory services, and financing affordable laboratory services.

Both technical and administrative laboratory leadership are required to provide direction in the development of the laboratory network implementation plan. Effective leadership must be in place to direct the development of the laboratory network and assure implementation of the Strategic Plan. The Director of Laboratory Services in the Ministry of Health must have adequate authority and resources to implement the plans. An organisational chart for laboratory services should clearly define roles and responsibilities. Initially, there must be a thorough assessment of the existing national laboratory network, and a technical working group should develop a thorough description of the current structure, practices, and functions of the laboratory system. The assessment should address laboratory infrastructure, equipment, staffing profiles, type of tests performed at each level, quality assurance systems, reporting, supervision and sources of funding. Efforts spent in documenting these practices, including what works or does not work in each of the assessment domains, will ensure a systematic approach to improving deficient areas and building a solid foundation for a national laboratory network.

In collaboration with stakeholders and public health programmes, the laboratory leadership group needs to assess the priority diseases, conditions and events that require laboratory confirmation and that are relevant to the country. Consideration should be given to the 10 core tests defined in the Global Health Security Agenda National Laboratory System Action Package.^12^ Diseases that are top causes of mortality/morbidity in the country and have epidemic potential should be included. In addition, the leadership group would determine the individual patient care needs that require the support of clinical laboratory testing. These are the tests typically performed in association with hospital inpatient or outpatient visits. Consideration should also be given to laboratory testing required for the clinical management of patients during potential epidemics, such as an outbreak of cholera or Ebola virus disease. The working group would suggest which testing can be performed in the tiered network within the country and which testing must be sent to sub-regional, regional or global reference laboratories. A plan addressing the geographic coverage needs for laboratory testing, as well as the test menu, should be developed that is specific to each country. The group would also ensure the contribution of laboratory services to universal health coverage at all levels of the national health system.

### Core capacities and infrastructure required to support effective laboratory networks in Africa

National laboratory networks must have core capabilities and adequate infrastructure in a number of important areas to function efficiently. The areas discussed below must be addressed in order to have a functional resilient laboratory network.

#### Universal health coverage

It is crucial for each country to ensure that its national laboratory system is part of its universal health coverage. In fact, there is an urgent need to re-examine the approach to formulating essential primary healthcare (district) laboratory packages to address health problems in different local contexts, and to re-define issues of access, quality and how public laboratory services should be funded and sustained.

#### Governance and organisation

To assure national ownership and leadership, governments should establish a department of laboratories within the Ministry of Health (Maputo Declaration)^2^ and appoint a National Director of Laboratories who oversees all the laboratories in the country, including public, clinical and private laboratories, as recommended by the 2008 WHO AFRO *Guide for national public health laboratory networking to strengthen integrated disease surveillance and response*.^18^ The Director must have the necessary authority and be able to communicate with the appropriate levels of the national health system as seen in a variety of countries, including Senegal and Burkina Faso. The Director assures that the private and public laboratories in the network are providing adequate service levels and meeting all standards for laboratory quality and safety. The National Director for Laboratory Service is directly linked to and coordinates activities with other health sector programmes. The organogram for national laboratory services must be defined with identification of all management roles and responsibilities for the network and their relationships with other programmes.

#### Tiered laboratory network structure

The structure of the tiered laboratory network must be established, taking into consideration the geographical distribution of the population in order to place laboratories in appropriate areas to assure patient care coverage. This network should be able to provide adequate laboratory testing coverage for > 95% of the population in order to meet individual patient care and public health needs. The distribution of laboratories in the country should be defined to match the distribution of the population. Private- and public-sector laboratories should be included in the network. The network or national public health laboratory, in collaboration with other sectors, such as food and agriculture, should lead all laboratory functions covered by the One Health Initiative.^19^ The network must be integrated with the surveillance and public health activities of the country. Channels of regular communication and specimen referral must be defined within and outside the network to assure maximum capacity to perform efficient testing. Working relationships must exist between laboratories at the local, regional and international level. In addition, the network should be fully integrated into public health outbreak response protocols and surge capacity must be available.

#### Well-designed, safe laboratory facilities

Laboratories must have a safe and suitable physical environment with appropriate space, power, climate control, water, internet/communication system, and transport access. There should be an uninterruptible power supply supporting laboratory equipment in case of power surges. Sufficient lighting, bench space, and clean water are also required. Biosafety Level 2, 3 or 4 requirements must be met depending on the types of testing that are to be performed. Current laboratories must be assessed for the adequacy of their physical infrastructure, including alignment with existing building construction codes and biosafety levels required for laboratory testing. Country standards should be defined for laboratory facility renovations and construction of new laboratories. An action plan to address laboratory facility upgrade needs should be developed and should include a prioritisation of facilities to be upgraded or built as part of the Strategic Plan. Adequate funding must be allocated for this upgrade plan by the Ministry of Health and/or other partners.

#### Supply chain management system

Laboratory testing requires the consistent availability of hundreds of types of consumable supplies and reagents. Supply chain management systems must exist at the central procurement and laboratory facility levels. The Director in the Division of Laboratories must actively collaborate with and inform procurement and central medical stores, so that laboratory needs are met. There must be adequate and efficient procurement and distribution systems to provide adequate supplies of reagents, consumables and quality control materials. Harmonisation of test equipment and assays will help limit the number of items that must be procured and kept in stock at all times. Supply consumption and forecasting systems must be developed and laboratory staff trained on supply inventory management to avoid stock-outs and excessive waste. An effective procurement system must be in place that will be able to maintain supplies in central stores for distribution to all sites. Surge capacity for reagents and supplies should be maintained. Efficient distribution systems to the various regions and facilities must be developed, and the laboratory environment should have adequate space to store cold chain and non-cold chain supplies.

#### In vitro diagnostic device regulation

The quality of reagents is essential for quality results; however, low-quality reagents are circulating in Africa. While the Director of Laboratories may not be directly responsible for this regulation, laboratory services is a major stakeholder. Unfortunately few countries have the capacity to review each in vitro diagnostic device (IVD) for quality, safety and performance in a pre-market setting. Baseline surveys of the IVD regulatory landscape in Africa in 2012 revealed that regulation of IVD is a neglected area.^20^ The Global Harmonization Taskforce model proposes a set of rules to place each IVD into a risk class dependent on the impact of the IVD on public and personal health.^21^ It is important to strengthen the capacity of countries for regulation of both pre- and post-market IVDs. Actions required include national IVD and medical device regulation policy, pre-marketing IVD registration, post-marketing surveillance and publication of a national authorised reagent list every year. In 2015, South Africa released draft regulations to address IVD issues.

#### Equipment management plan

Laboratory equipment must be procured, maintained, and regularly replaced to assure that laboratory testing is consistently available at all sites within the network. An equipment management plan should be developed that lists the equipment required for each tier of the laboratory network. Equipment should be harmonised and appropriate for the complexity of the laboratory at each tier in order to reduce the training, maintenance, and supply chain burdens for the country. Harmonisation also allows for easy referral of specimens to other network laboratories, and makes the job of developing standard operating procedures, training, competency assessment, and reference intervals easier. This plan must be updated annually. All decisions on equipment for the network should be made in consultation with the Director of the Division of Laboratories. The network should have an equipment manager to support management of all equipment placed in laboratories. A plan for equipment maintenance must be developed that assures responsive and efficient preventative maintenance (e.g., training of lab personnel) and corrective maintenance (e.g., recruitment of competent maintenance technicians, maintenance contracts with outside vendors). Back-up analysers or a good specimen referral system should be in place to assure critical testing needs are met on a continuous basis.

#### Quality systems management

The elements of an effective laboratory quality system include organisation, standard operating policies and procedures, a quality control system, planned quality improvement activities, external quality assessments, document control, and a plan for accreditation. These are described in detail below:

1. **Organised quality unit:** An organised quality unit reporting directly to the Director of Laboratory Services must exist to develop and manage all aspects of the quality system in the country, including the external quality assessment system. Laboratory quality officers should be identified at each laboratory to assist with quality system implementation. Regular supportive supervision by quality unit staff or quality officers from a higher tier laboratory is required to assure that all elements of the quality system are in place in each laboratory. These visits provide mentoring and training of quality officers and laboratory staff on quality systems and test performance.
2. **Standard operating procedures:** Standard operating procedures must be understood by staff and implemented to ensure overall test reliability, which includes accuracy and precision. After equipment and testing methods are determined and harmonised, standard operating procedures should be developed by the quality unit at the central level for all laboratories in the network. These standard documents can serve as templates for all laboratories and be modified to meet the needs of individual laboratories. This reduces the amount of time spent on developing standard operating procedures at each laboratory in the network. Experts in the equipment system or assay are usually the best persons to develop the standard operating procedures for dissemination to the other network laboratories. A document control system must be in place for all laboratory documents.
3. **Quality control:** Laboratory professionals must routinely perform and evaluate quality control samples to guarantee that test methods and equipment perform according to the established standards. Quality control procedures should be standardised based on equipment and methods in use. Quality control materials must be of high quality and available continuously. Since these materials may have a short shelf life, special procurement methods must exist to assure continuous availability. Patient testing should not be released by staff without running quality control according to standard operating procedures. Quality control results must be reviewed regularly by laboratory supervisors to assure quality control procedures are run and corrective actions are taken when results do not meet standards.
4. **External quality assessment plan:** An external quality assessment plan should be developed by the quality unit to specify the scope and participation of laboratories in the network. The plan should address the national external quality assessment requirements, including types of programmes required for each laboratory in the network. Laboratories must participate in external quality assessment programs for critical assays in microbiology, haematology, immunology, microscopy and chemistry, in order to demonstrate that the laboratory has an acceptable level of quality. Definitions of acceptable performance in external quality assessment programmes should be defined and reports available to all laboratories. Laboratories must evaluate external quality assessment results and take appropriate corrective actions. The source of external quality assessment programmes (national or international) for each test should be defined in the plan. In order to reach all statewide laboratories, a national external quality assessment programme can be run by national health or reference laboratories (e.g., Burkina Faso).^22^ National health or reference laboratories should participate in an international external quality assessment programme.
5. **Accreditation plan:** A plan for accreditation of laboratories within the network by the national laboratory should exist using the WHO Stepwise Laboratory Quality Improvement Process Towards Accreditation checklist^23^ and/or an international accreditation programme. The plan should define the type of accreditation planned for all laboratories in the network. National reference laboratories should try to achieve international accreditation. A timeline and roadmap for accreditation activities would be developed by the quality unit.

#### Workforce development

The country should possess a human resource development strategy for the laboratory sector. Job descriptions should be available that define roles, responsibilities and qualifications for the entire network. Adequate numbers of well-trained, competent and motivated laboratory scientists and technologists/technicians must be available in-country to manage the laboratories in the network and to perform routine and specialised testing. Strong pre-service curricula must be in place to train laboratory workers entering the field at diploma and/or degree levels. Competency-based certification to perform tests within their scope of duties must be completed for all staff. National licensing or registration systems for laboratory staff would be ideal. Opportunities for continuing professional development must exist to develop management, leadership, and higher level technical skills required by the network. These skills must exist to provide the required technical and administrative oversight of the laboratory network. Programmes such as Strengthening Laboratory Management Towards Accreditation are available to strengthen core management skills so that staff can manage technical operations and achieve accreditation.

#### Laboratory staffing

Appropriate workload-based staffing models should be developed for each laboratory in the system to assure adequate numbers of staff and correct skill mix. Skill levels of testing personnel must be tied to the complexity of instrumentation and methods in use at each tier. Once the staffing model is developed, an assessment must be made of the adequacy of technical staff in the country to meet the projected needs. Plans will need to be developed to address the recruitment of the required numbers of laboratory technicians, technologists, scientists, and maintenance technicians. Strategies must be developed to retain competent staff in laboratories such as career paths and adequate remuneration and incentives.

#### Information management and communication systems

A system of immediate and regular reporting of district laboratory information to the provincial and to the national levels, as well as to patient care providers, would be expected. Laboratories require adequate computer systems, internet access, and other electronic devices in order to transmit reports and data within the country. Laboratories generate information that must be captured and transmitted either on written reports or electronically in a laboratory information system. Within a national health system, a hospital information system with a laboratory component may be desirable to transmit laboratory orders and results electronically. The ability to meet patient care needs, integrated disease surveillance needs and laboratory testing/reporting with one health sector computer system will be most efficient and cost-effective for the country. An alternative is a laboratory information management system that interfaces with a variety of other systems/databases in the country. The laboratory information management system must be capable of generating a variety of management reports that provide data for public health surveillance and laboratory monitoring and evaluation purposes. These reports should be able to easily pull antimicrobial resistance and reportable disease data and reports. Standardised laboratory request forms and public health report forms can be used if information systems are not available. The presence of paper forms makes email and fax capability extremely important in order for referral systems to return laboratory results back to the site of patient care and to public health authorities. Free access laboratory information management system could be developed and installed in laboratories to ease the work of technicians and facilitate the transfer of epidemiological data (e.g., Labbook used by West African Laboratory Network). It is also necessary to develop an information technology network infrastructure to support any laboratory computer system.

#### Specimen collection, referral and transport

Laboratories at peripheral sites in the network will usually have a more limited test menu. Health facilities must have the ability for trained staff to collect and properly package specimens at one location and transport them to the next level of a tiered network for testing, without compromising the quality of the specimens or the safety of the packaging and transport staff. These specimen referral and transport systems should be integrated to include all specimens, where possible – at minimum, systems should at least be coordinated. Adequate tracking and chain of custody systems must exist and standard operating procedures should exist for all specimens being referred. The ability to safely collect, package and transport specimens to another laboratory, as well as to get laboratory reports back to the originating facility or to public health authorities in a timely manner, is critical. Special packaging guidelines published by the International Air Transport Association are required for highly pathogenic materials. Adequate transportation systems covering all necessary routes need to be defined for these functions. The utilisation of other existing logistics systems within the laboratory network, broader health system or private sector should be explored.

#### Biologic risk management, including waste management

Biologic risk management includes laboratory biosafety and biosecurity, which share the common goal of safe handling of biologic materials and keeping valuable biologic materials safe and secure during use, storage, and transport.^23^

**Biosafety.** Biosafety guidelines should be developed to establish safe work practices and containment levels for all laboratories. Biosafety risk assessment must be done at each facility to determine the biologic risks that will be encountered in the laboratory. A determination of the biosafety level for each facility based on type of testing should guide the type of protective equipment and containment equipment required. Biosafety guidelines should address all aspects of safety in the laboratory, including personal protective equipment, engineering controls, phlebotomy safety, sharps precautions, post-exposure prophylaxis, and waste management. In addition any special precautions unique to the type of pathogens being tested must be implemented. Laboratories may reference the WHO *Laboratory Biosafety Manual*, 3rd edition, for biosafety recommendations for laboratories.^24^ All laboratory staff must be trained on biosafety precautions specific to their work. Laboratory safety audits should be performed by quality or biosafety officers to assure compliance with biosafety guidelines.

**Biosecurity.** Countries will need to adopt national legislation for management of biosecurity and biosafety risks.^25^ These regulations would address the use and control of dangerous pathogens domestically, as well as measures to control the export of these pathogens. A biosecurity plan should be developed by the Division of Laboratories to establish accountability for the secure handling, storage and disposal of valuable biologic materials, such as patient specimens, reference strains, microbiologic cultures or dangerous pathogens. An initial risk assessment would be performed by laboratory management and scientific staff to determine the level of biosecurity measures needed based on the country’s needs. A distinction must be made between specimens and materials that require additional biosecurity measures and those that will be tested on a one-time basis, stored briefly then destroyed by the laboratory. Standard operating procedures must be developed for handling and storage of routine specimens as well as valuable biologic materials, including dangerous pathogens. Biosecurity plans will need to address all items listed in Box 1.

BOX 1 Biosecurity plan features.

**Table:** 

Identification of materials that will be subject to biosecurity accountability practices
Defined roles and responsibilities for implementing the biosecurity plan
Maintenance of regularly updated inventories of dangerous pathogens and valuable biologic material being stored at each facility
Restricted access to laboratories including specimen storage areas. Defined storage location for routine laboratory specimens and for VBM.
Specific control measures to track and document the inventory, use, production, transfer and destruction of defined materials
Procedures for the appropriate intake, storage, use, transfer, and destruction of defined materials
Specific procedures for the transfer of VBM between facilities and outside of the country (chain of custody forms)
Certification of the inactivation or destruction of defined materials
Restricted access to defined materials by authorised personnel and documentation of all personnel accessing materials
Training of all laboratory staff on the biosecurity measures for the laboratory
Biosecurity programme audits should be conducted to assure compliance

VBM, Valuable Biologic Materials.

BOX 2 Lessons Learned.

**Table:** 

Functional laboratory networks require a robust workforce, strong leadership and significant financial investment to be successful.
National laboratory networks must be closely integrated with the country’s public health system and include all types of laboratories in the public health system.
A strong, well-resourced national public health laboratory is required for development of the network.
Significant core capabilities and infrastructure must be developed and maintained for laboratory networks to be functional.

#### Priority disease surveillance

A priority list of communicable diseases and dangerous pathogens should be used to determine laboratory service requirements for the country. National laboratory network laboratories should be able to conduct testing to support integrated disease surveillance for at least 10 priority infectious diseases, as described earlier. This requires an integrated, tiered laboratory network with modern diagnostic capabilities and real-time surveillance capabilities. Data from laboratory test systems should be available electronically in real-time to all stakeholders in the public health sector through interconnected information systems or databases. Laboratory-based surveillance must be fully implemented with a national reporting system for antimicrobial resistance. One reference laboratory in the country must be able to identify three of the seven WHO priority antimicrobial resistance pathogens. Ideally, laboratories at regional and national levels should be able to identify pathogens and perform antibiotic susceptibility testing. A surveillance system should also be in place for the detection of three to five priority zoonotic diseases. In order to accomplish this, multi-sector collaboration must exist between the laboratory network and the agriculture sector.

### Conclusion

Successful laboratory networks are the direct result of effective laboratory leadership, accurate assessment, strategic and operational planning, and significant financial investment, particularly in human resource capacity. The development of successful laboratory networks begins with a baseline assessment followed by a good strategic planning process. Laboratory leadership must be empowered and provided with the resources to plan, develop and monitor the laboratory network to meet the public health needs of the country. Each of the core capabilities described here must be systematically planned and implemented to assure a functional national laboratory network. The integration of the laboratory network with the public health system of the country is required to meet current and emerging disease threats in the era of global health security.
